# Applying the extended health belief model to understand salt-reduction behaviors among Saudi adults: a cross-sectional study

**DOI:** 10.3389/fpubh.2026.1836238

**Published:** 2026-06-15

**Authors:** Haya Aljadani, Buthaina M. Aljehany, Eman A. Abduljawad, Sara A. Alasmari, Rowida Khader Allily, Nada Benajiba

**Affiliations:** 1 Food and Nutrition Department, Human Sciences and Design Faculty, King Abdulaziz University, Jeddah, Saudi Arabia; 2 Public Health Department, College of Health Sciences, Saudi Electronic University, Riyadh, Saudi Arabia; 3 Joint Research Unit in Nutrition and Food, RDC-Nutrition AFRA/IAEA, Ibn Tofail University-CNESTEN, Kenitra, Morocco; 4 College of Health Sciences, International University of Rabat, Rabat, Morocco

**Keywords:** adults, dietary practices, extended health belief model, knowledge, salt intake, Saudi Arabia

## Abstract

**Background:**

High sodium intake is a major risk factor for hypertension and cardiovascular disease, with consumption levels in Saudi Arabia far exceeding global recommendations. Understanding the psychosocial determinants of salt-related behaviors is essential for designing effective interventions.

**Objective:**

This study applied an extended Health Belief Model (HBM) to examine the knowledge, perceptions, and habitual practices related to salt intake among Saudi adults.

**Methods:**

A cross-sectional study was conducted with 522 Saudi adults using a translated and validated, self-administered questionnaire assessing sociodemographics, salt-related knowledge, HBM constructs (perceived susceptibility/severity, risk, benefits, barriers, cues to action, self-efficacy), and dietary practices.

**Results:**

While participants demonstrated moderate to high awareness of the health risks of high salt intake (e.g., 85.1% linked it to hypertension), technical knowledge was limited, with only 15.3% correctly identifying the World Health Organization’s daily salt recommendation. Habitual salt use during cooking was high (55.2% always), and consumption of salty snacks and fast food was frequent. Dietary practices were suboptimal across all groups. Significant positive associations were found between dietary practices and behavioral intention (*r* = 0.291), self-efficacy (*r* = 0.248), and cues to action (*r* = 0.234). A persistent awareness-behavior gap was observed, where favorable perceptions did not translate into consistent salt-reduction actions.

**Conclusion:**

Saudi adults demonstrate moderate to high awareness of salt-related health risks but limited technical knowledge and low adoption of salt-reduction behaviors. The findings suggest that behavioral intention, self-efficacy, and cues to action are associated with more favorable salt-reduction practices. Targeted, multifaceted interventions addressing cognitive, skill-based, and environmental determinants are essential to reduce salt intake, prevent hypertension, and support national and global health objectives.

## 1 Introduction

Salt, commonly known as the “master of taste,” serves essential functions in food preparation by intensifying flavor profiles, preserving food quality, and modifying sensory attributes such as appearance and consistency (1–3). From a physiological perspective, sodium chloride is indispensable for maintaining fluid homeostasis, regulating pH levels, and supporting various metabolic processes (4). However, the global mean sodium intake is estimated to be 4,310 mg per day (10.78 g of salt per day), which far exceeds the physiological requirement and is more than double the World Health Organization recommendation of fewer than 2,000 mg of sodium (equivalent to less than 5 g of salt) per day in adults (5). This overconsumption has significant public health implications, with epidemiological studies linking high sodium intake to millions of preventable deaths annually (6). Indeed, scientific evidence demonstrates that excessive sodium consumption is strongly correlated with hypertension and cardiovascular disorders (7), as well as other chronic conditions, including type 2 diabetes, obesity, cerebrovascular accidents, renal calculi, and reduced bone density (8–10). In response to this growing health crisis, the World Health Organization has proposed a global initiative to reduce sodium intake by 30% by 2025, a strategy projected to prevent approximately 7 million premature deaths by 2030 (5).

In Saudi Arabia, hypertension affects approximately 9.2% of individuals aged 15 years and older (11). Despite growing awareness of its risks, average sodium intake among Saudis remains high, ranging between 9 and 12 grams per day—more than double the World Health Organization’s recommended limit of 5 g/day (12). Among university students, for instance, Al Khathaami et al. (13) reported that 97.8% exceeded the recommended salt intake, with a mean daily consumption of 6.76 g. Although excessive salt consumption is prevalent, knowledge about salt and its health risks remains limited (14) and does not appear to influence actual dietary behaviors significantly. In a cross-sectional study of female students, 70% reported using table salt regularly, further highlighting the gap between awareness and practice (15). To address such challenges, the Saudi Food and Drug Authority (SFDA) has implemented front-of-pack labeling (FOPL) initiatives, including the traffic light labeling system, to guide healthier food choices (16). According to the regional review by Al-Jawaldeh et al. (17), the overall compliance rate in Saudi Arabia was 47%, with the highest compliance observed in products such as pasta (100%), butter (88%), cooking sauces (92%), and pizza (80%). In contrast, low compliance rates were reported for ready-made meals (0%), canned vegetables (12%), and cheeses (22%). While such regulatory efforts are commendable, their effectiveness depends on individual behavior change, which in turn is influenced by psychosocial factors (18).

To date, no study in Saudi Arabia has applied a behavioral framework—such as the Health Belief Model (HBM)—to examine the determinants of salt-related practices. The HBM offers a robust theoretical lens for understanding how perceived susceptibility and severity to diseases such as hypertension, perceived severity, perceived benefits and barriers to reducing salt intake, and self-efficacy shape health behaviors (19). This study seeks to fill that gap by applying the HBM to examine how Saudi adults’ beliefs, cultural norms, and socio-demographic factors influence their salt intake. Findings aim to support the design of culturally sensitive, evidence-based interventions that align with the goals of Saudi Vision 2030 to reduce the burden of non-communicable diseases and promote healthier lifestyles (20).

### 1.1 Theoretical framework

The HBM provides a comprehensive framework for understanding the psychosocial determinants of health-related behaviors, including dietary salt intake reduction. According to the HBM, individuals are more likely to adopt preventive behaviors when they perceive themselves to be at risk of a health condition such as hypertension (perceived susceptibility) and believe that the condition may have serious consequences (perceived severity) (21, 22). Furthermore, individuals must perceive that the benefits of adopting a behavior—such as reducing salt intake—outweigh the perceived barriers, which may include taste preferences, social influences, or limited availability of low-salt options (21, 23, 24). To enhance its explanatory capacity, the model has been extended to include self-efficacy, defined as the individual’s confidence in their ability to perform the behavior, and cues to action, which represent internal or external triggers such as medical advice, family encouragement, or media exposure (19, 25).

In the context of salt-reduction behaviors among Saudi adults, this extended HBM is further enriched by incorporating salt-related knowledge, which plays a critical role in shaping individuals’ perceptions and behavioral intentions. Higher levels of knowledge regarding recommended salt intake, health risks, and preventive strategies have been associated with improved dietary practices and greater motivation to reduce salt consumption (26, 27). Similarly, a recent cross-sectional study conducted among healthy UAE residents demonstrated significant associations between salt-related knowledge, attitudes, and practices and objectively measured 24-h urinary sodium and potassium excretion, highlighting the relevance of KAP factors in shaping sodium-related behaviors within Gulf populations (28). Additionally, sociodemographic and health-related characteristics—including age, gender, marital status, education, income, body mass index, presence of chronic diseases, and family history of hypertension—may influence both knowledge and health beliefs, thereby indirectly affecting behavior (24, 29). Exposure to health information through physicians, family members, and media sources also serves as important cues that may facilitate behavior change.

**Figure 1:**
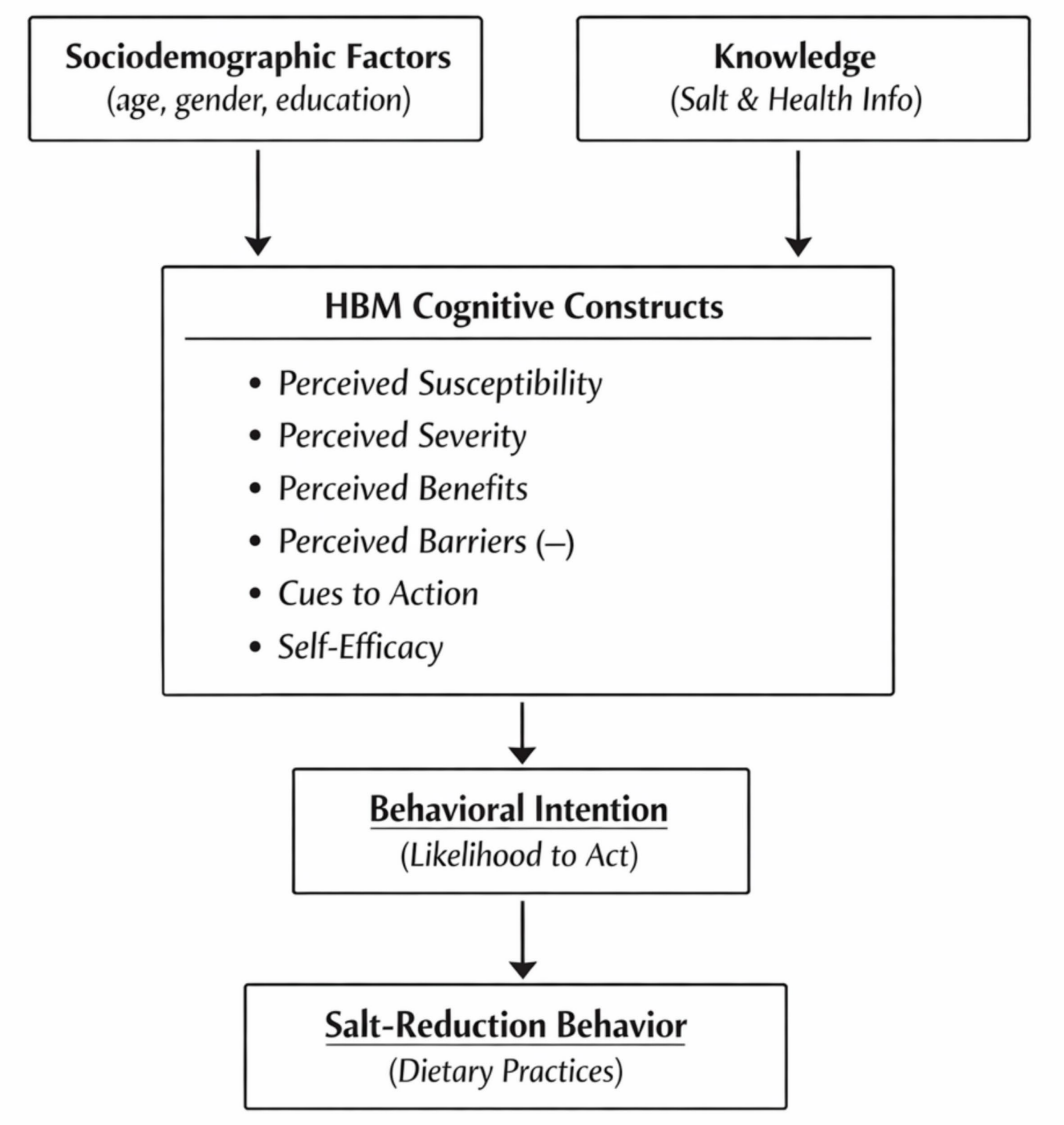
Extended health belief model for salt-reduction behaviors.

Based on this framework, dietary practices related to salt intake reduction are conceptualized as the behavioral outcome of interest, influenced by cognitive (HBM constructs), informational (knowledge), and contextual (sociodemographic and environmental) factors. In particular, perceived susceptibility, perceived severity, perceived benefits, cues to action, self-efficacy, and the likelihood of following recommended interventions (behavioral intention) are expected to positively influence salt-reduction behaviors, while perceived barriers are expected to hinder such practices. This integrated model reflects a multidimensional understanding of behavior, acknowledging that adopting and sustaining salt-reduction practices requires not only awareness and motivation but also confidence and supportive environmental conditions (Figure 1).

## 2 Materials and methods

### 2.1 Study population and study design

This study adopted a cross-sectional design and was conducted across all regions of Saudi Arabia over 6 months. Eligible participants were Saudi citizens aged 18 years or older who currently reside in Saudi Arabia. Individuals were excluded if they were pregnant, lactating, or following a medically prescribed diet (e.g., for renal disease), as their behaviors may already be influenced by clinical advice.

Ethical approval for this study was obtained from the Biomedical Ethics Research Committee at King Abdulaziz University (Reference No 341–25). Participation in the study was entirely voluntary and posed no physical or psychological risk to participants. All individuals were provided with an informed consent form that clearly outlines the study’s purpose, procedures, potential risks and benefits, and the rights of participants, including the right to withdraw at any time without consequence. Informed consent was obtained electronically on the first page of the questionnaire. No personally identifiable information was collected. All responses were strictly anonymous and confidential, and the data were used exclusively for academic research purposes. This process ensures full adherence to ethical research standards and the protection of participants’ rights and privacy.

### 2.2 Sampling technique and sample size calculation

Participant recruitment was conducted using an online self-administered questionnaire hosted on Google Forms. The electronic survey was disseminated via popular social media platforms, including WhatsApp and Telegram. As such, a non-probability convenience sampling technique was employed. The sample size was calculated using a standard formula for non-probability sampling, assuming a prevalence rate of 50%, given the absence of national data on the proportion of Saudi adults who engage in salt-reduction behavior. Based on the estimated adult population of approximately 13 million in Saudi Arabia, as reported by The Saudi Census (30), the required minimum sample size is 385 participants. To account for potential incomplete responses, an additional 10% was added, resulting in a final target sample size of 422 participants.


$$n=\frac{Z_{\alpha /2}^{2}P(1-P)}{E^{2}}$$

n = required minimum sample size.

P = the estimated prevalence of an indicator.

*α* = the level of significance.

Zα = the z-score corresponding to the degree of confidence.

E = Desired precision.

To enhance data quality and ensure the consistency of participant responses, multiple quality assurance measures were applied. The online survey was designed to restrict participants to a single submission through IP and account-based controls, thereby minimizing duplicate entries. Furthermore, responses were reviewed for completeness and for response patterns such as straight-lining, where identical answers were selected across Likert-scale items, before being included in the final analysis. Participants involved in the pilot testing phase were excluded from the final analytical sample to avoid potential response bias.

### 2.3 Study tool

The study instrument consisted of a structured, self-administered questionnaire divided into the following four sections:

Section 1: Sociodemographic data: This section collected information on participants’ age, gender, marital status, region of residence, education level, employment status, monthly income (in Saudi Riyals), and self-reported height (m) and weight (kg), as well as the presence of any chronic health conditions. According to the World Health Organization (31), the body mass index (BMI) was calculated as weight in kilograms divided by the square of height in meters (kg/m^2^) and categorized as follows: underweight (<18.5 kg/m^2^), normal weight (18.5–24.9 kg/m^2^), overweight (25.0–29.9 kg/m^2^), and obesity (≥30.0 kg/m^2^). In addition to these core variables, participants were asked about whether they have relatives diagnosed with hypertension, and whether they have ever been advised by a physician, or encouraged by family members, relatives, or friends to reduce their salt intake. They were also asked whether they had encountered media-based advertisements or educational materials (e.g., on television, in magazines, online, or in books) emphasizing the importance of reducing dietary salt intake.

Section 2: Assessment of Salt-Related Practices: Salt-related behaviors were assessed through 10 positively worded items adapted from Cheikh Ismail et al. (27) and Grimes et al. (32), with a total possible score ranging from 10 to 50. Each item was rated using a five-point Likert scale: “never,” “rarely,” “sometimes,” “usually,” and “always.” The items addressed various dimensions of salt consumption, including the frequency of adding salt during cooking, using salt at the table, and consuming salty foods, snacks, and seasonings such as fast food, pickles, sausages, salty snacks, and dried salty foods. For these first five items, higher scores (i.e., 5 for “never” and 1 for “always”) indicated more desirable salt-reduction behaviors. The remaining five items focused on specific salt-reduction strategies such as making efforts to reduce their salt intake and whether they are using salt substitutes, reading food labels to check for salt or sodium content, purchasing no-salt or reduced-salt food products, and requesting that meals be prepared without added salt when eating out. For these items, the scoring was straightforward, with 1 point for “never” and 5 points for “always,” indicating increasingly health-conscious behaviors.

Section 3: Knowledge Related to Salt and Hypertension: This section assessed participants’ knowledge of health risks associated with high salt intake through 10 categorical questions, adopted from Alhazmi et al. (26) and Cheikh Ismail et al. (27). The items address the recommended daily salt intake, diagnostic criteria for hypertension, the health effects of excessive salt consumption, and salt-related preventive strategies. Questions cover links between salt intake and conditions such as hypertension, cardiovascular and kidney diseases, obesity, and complications of uncontrolled blood pressure. Participants were also asked to define salt-rich foods and whether reducing salt intake improves health. Each item includes response options, including “do not know.” Responses were scored as 2 points for a correct answer and 1 point for an incorrect or “do not know” answer. Thus, the possible score for this section ranged between 10 and 20 points.

Section 3: This section evaluates participants’ perceptions and behavioral intentions regarding salt-reduction practices, using a translated into Arabic and validated Determinants of Salt-Restriction Behavior Questionnaire (DSRBQ), originally developed by Chan et al. (33). This instrument is theoretically grounded in the HBM and has been culturally adapted for relevance to the Saudi population. It is designed to capture a wide range of cognitive, emotional, and behavioral factors that influence individuals’ willingness and ability to reduce dietary salt intake.

Participants responded to 36 items distributed across seven subscales based on the HBM, using a 5-point Likert scale ranging from 1 (strongly disagree) to 5 (strongly agree). Each subscale represented a core HBM construct, and scores were calculated by summing item responses, with higher scores indicating stronger endorsement of the respective construct. Perceived susceptibility and severity of the disease (5 items; score range: 5–25) assessed beliefs about personal risk of developing diseases such as hypertension and their potential severity (6 items; score range: 6–30) measured perceptions of the seriousness and consequences of high blood pressure, including physical, financial, and social impacts. Perceived benefits (3 items; score range: 3–15) evaluated beliefs regarding the effectiveness of a low-salt diet in preventing or managing hypertension, whereas perceived barriers (8 items; score range: 8–40) captured cultural, practical, and psychological challenges to reducing salt intake, such as taste preferences and family resistance. Cues to action (6 items; score range: 6–30) reflected exposure to internal or external triggers, including health advice, media messages, or social encouragement, that may prompt behavior change. Self-efficacy (3 items; score range: 3–15) assessed confidence in the ability to adopt salt-reduction behaviors in different contexts, and the likelihood of following recommended interventions (7 items; score range: 7–35) represented participants’ intention to implement and sustain salt-reduction strategies despite potential barriers.

### 2.4 Translation, adaptation, validity, and reliability of the questionnaire

This study employed an adapted Arabic version of the Determinants of Salt-Restriction Behavior Questionnaire (DSRBQ) originally developed by Chan et al. (33) to assess knowledge, perceptions, and behaviors related to salt intake and hypertension prevention. The original English instrument was translated into Standard Arabic and culturally adapted to reflect commonly consumed foods and lifestyle patterns relevant to the Saudi context. The translation and adaptation process followed established best-practice guidelines for cross-cultural instrument development, including forward translation, back-translation, and reconciliation. Specifically, a panel of bilingual nutrition experts independently translated the questionnaire into Arabic with a focus on conceptual rather than literal equivalence. The translated version was then back-translated into English by independent bilingual professionals unfamiliar with the original instrument to ensure semantic accuracy. Discrepancies between the original and back-translated versions were reviewed and resolved through consensus discussions within the research team, resulting in a culturally appropriate and linguistically valid Arabic version of the questionnaire (34).

Following translation, the instrument underwent rigorous validity assessment to ensure its suitability for the target population. Face validity was evaluated by an expert panel using criteria related to content completeness, comprehensibility, and appropriateness of completion time, in line with established recommendations (35). Content validity was assessed by having experts rate the relevance of each item on a 4-point scale, and the Content Validity Index (CVI) was subsequently calculated at both the item and scale levels following the methodology described by Rodrigues et al. (36). The results demonstrated strong validity, with item-level CVI (I-CVI) values ranging from 0.80 to 1.00, and scale-level CVI (S-CVI/Ave) reaching 0.981, exceeding the recommended threshold of 0.90. Additionally, the universal agreement index (S-CVI/UA) was 0.885, surpassing the acceptable minimum of 0.80, indicating a high level of expert agreement regarding item relevance. These findings confirm that the adapted questionnaire is both valid and appropriate for assessing salt-related knowledge and behaviors in the Saudi population.

To evaluate the internal consistency and stability of the questionnaire, a pilot study was conducted among at least 50 participants who met the inclusion criteria. All participants completed the questionnaire independently without assistance, ensuring standardized administration conditions. Internal consistency reliability was assessed using Cronbach’s alpha coefficient for each domain of the instrument, which is widely recommended for evaluating the homogeneity of multi-item scales (37). A threshold of *α* ≥ 0.70 was considered acceptable, consistent with established psychometric standards. Where necessary, items were reviewed and refined based on the obtained reliability coefficients, and the revised version of the questionnaire was subsequently re-tested using the same methodology to ensure improved internal consistency.

The results demonstrated high internal consistency across all domains of the questionnaire, with Cronbach’s alpha values ranging from 0.76 to 0.98. Specifically, the perception and knowledge domains showed strong reliability (α = 0.87 and α = 0.85, respectively). The overall HBM construct exhibited excellent internal consistency (α = 0.96), with subscale values also indicating good to excellent reliability, including perceived susceptibility (α = 0.76), perceived severity (α = 0.92), perceived benefits (α = 0.98), perceived barriers (α = 0.91), cues to action (α = 0.89), and self-efficacy (α = 0.88). The recommended interventions domain also showed excellent reliability (α = 0.96). These findings indicate a high level of consistency among questionnaire items, suggesting that the instrument is reliable for assessing salt-related knowledge, perceptions, and behaviors in the target population.

### 2.5 Data analysis

Data were analyzed using the SPSS software, Version 26.0. Descriptive statistics, including means, standard deviations, frequencies, and percentages, were used to summarize participants’ demographic characteristics, as well as mean knowledge scores related to salt consumption and hypertension risk. The mean score for each HBM construct and dietary practices for each participant was calculated by dividing the total obtained score by the number of items. Pearson correlation coefficients (r) were computed to explore associations between continuous variables, such as age, BMI, salt-related behaviors, and HBM constructs. Independent t-tests were used to examine differences in perceptions and behaviors across binary variables such as gender and age groups. For multi-category variables, one-way ANOVA was conducted to examine group differences, with normality and homogeneity of variances verified prior to analysis. Non-parametric alternatives were considered, but data met the assumptions for parametric testing. No post-hoc comparisons were conducted, as the study aimed to detect overall group differences rather than identify specific pairwise distinctions. A 95% confidence interval (CI) was applied, and statistical significance was set at *p* ≤ 0.05.

## 3 Results

### 3.1 General characteristics of the studied population

The study included 522 Saudi adults (Table 1), predominantly female (73.6%). Most participants were young to middle-aged, with 60.8% between 18 and 39 years. In terms of BMI, while 35.2% had normal weight, 31.8% were overweight. More than half of the participants were married (58.6%), and nearly half (49.6%) were from the Western region of Saudi Arabia. Educational attainment was high, with 79.6% holding a university degree or postgraduate qualification. Regarding income, 44.4% reported a monthly household income below 5,000 SAR.

**Table 1:** General characteristics of the studied population (*n* = 522).

Characteristics	N	%
Gender		
Male	138	26.4
Female	384	73.6
Age		
18–29 years	192	36.7
30–39 years	126	24.1
40–49 years	114	21.8
50–59 years	90	17.2
Body mass index		
Underweight: < 18.5 kg/m^2^	32	6.1
Normal weight: 18.5–< 25.0 kg/m^2^	184	35.2
Overweight: 25.0– <30.0 kg/m^2^	166	31.8
Obesity: ≥ 30.0 kg/m^2^	140	26.8
Marital status		
Married	306	58.6
Unmarried	216	41.4
Place of residence		
North	92	17.6
South	50	9.6
Central	63	12.1
East	58	11.1
West	259	49.6
Education level		
Primary education	9	1.7
Secondary education	97	18.6
University degree or diploma	327	62.6
Postgraduate studies	89	17.0
Occupation		
Private Sector	101	19.3
Public Sector	132	25.3
Self-Employed	16	3.1
Student	116	22.2
None	157	30.1
Household income (per Month in SAR)		
Less than 5,000	232	44.4
From 5,000 to 10,000	129	24.7
From 11,000 to 20,000	107	20.5
More than 20,000	54	10.3
Do you have relatives who suffer from high blood pressure?		
Yes	384	73.6
No	86	16.5
I do not know	52	10.0
Diagnosed with high blood pressure		
Yes	112	21.5
No	410	78.5
Doctor advised salt reduction		
Yes	163	31.2
No	302	57.9
I do not remember	57	10.9
Family/friends advised salt reduction		
Yes	306	58.6
No	183	35.1
I do not remember	33	6.3
Seen advertisements about salt reduction		
Yes	344	65.9
No	112	21.5
I do not remember	66	12.6

In terms of hypertension-related characteristics, 21.5% reported being diagnosed with high blood pressure, and 73.6% had relatives with hypertension. About one-third (31.2%) had been advised by a doctor to reduce salt intake, while 58.6% had received advice from family or friends. Additionally, 65.9% reported having seen advertisements about salt reduction.

### 3.2 Habitual salt consumption behavior

Table 2 shows the habitual salt consumption behavior by participants. More than half of participants (55.2%) reported always using salt during cooking, and an additional 25.3% reported often doing so. In contrast, discretionary salt addition at the table was less common, with 36.8% reporting they never add salt to food at the table. Consumption of salty foods was frequent. Nearly half of participants reported sometimes to always consuming salty processed foods such as pickles and sausages (60.2%), salty snacks (75.5%), and salty fast food (66.8%), indicating substantial exposure to hidden dietary salt sources.

**Table 2:** Habitual salt consumption behaviors among the studied population (*n* = 522).

Habitual salt consumption behaviors	N (%)				
	Never	Rarely	Sometimes	Often	Always
I use salt during cooking	15 (2.9)	19 (3.6)	68 (13.0)	132 (25.3)	288 (55.2)
I add salt to food at the table	192 (36.8)	135 (25.9)	96 (18.4)	51 (9.8)	48 (9.2)
I consume salty foods such as pickles, sausages, and soy sauce	66 (12.6)	142 (27.2)	205 (39.3)	69 (13.2)	40 (7.7)
I consume salty snacks such as salted nuts, salted dried vegetables, and salted chips	33 (6.3)	95 (18.2)	216 (41.4)	128 (24.5)	50 (9.6)
I consume salty fast food	47 (9.0)	126 (24.1)	196 (37.5)	103 (19.7)	50 (9.6)
I seriously try to reduce salt intake	79 (15.1)	87 (16.7)	190 (36.4)	101 (19.3)	65 (12.5)
I use salt substitutes such as natural seasonings or lemon juice	147 (28.2)	121 (23.2)	133 (25.5)	84 (16.1)	37 (7.1)
I read food labels to check the amount of salt/sodium	187 (35.8)	139 (26.6)	103 (19.7)	50 (9.6)	43 (8.2)
I buy low-salt or salt-free foods	134 (25.7)	141 (27.0)	144 (27.6)	75 (14.4)	28 (5.4)
I ask restaurants to prepare meals without salt	321 (61.5)	89 (17.0)	61 (11.7)	30 (5.7)	21 (4.0)

Regarding salt-reduction behaviors, only 31.8% reported often or always seriously trying to reduce salt intake. The use of salt substitutes was relatively low, with only 23.2% reporting often or always using alternatives such as natural seasonings or lemon juice. Label-reading behavior was also limited, as 62.4% reported never or rarely checking food labels for salt/sodium content. Similarly, purchasing low-salt or salt-free foods was inconsistent, and the majority (78.5%) never or rarely asked restaurants to prepare meals without salt.

### 3.3 Salt-related knowledge

Salt-related knowledge by the studied population is presented in Table 3. Most participants correctly recognized that high salt intake is a risk factor for hypertension (85.1%), cardiovascular disease (68.2%), kidney problems (79.5%), and that reducing salt intake lowers blood pressure and improves health (86.6%). A high proportion also identified fast foods as high in salt (84.5%) and acknowledged that uncontrolled hypertension can lead to serious complications such as stroke and heart failure (75.9%). However, knowledge of technical and guideline-based information was limited. Only 15.3% correctly identified the World Health Organization-recommended maximum daily salt intake for adults, with 64.0% selecting “do not know.” Similarly, only 10.5% correctly identified the threshold for classifying a food as high in salt, and 58.2% reported not knowing. Knowledge of hypertension diagnostic criteria was also suboptimal, with only 34.1% answering correctly.

**Table 3:** Salt-related knowledge by the studied population (*n* = 522).

Salt-related knowledge items	N (%)		
	Correct	Incorrect	Do not know
What is the World Health Organization’s recommended maximum daily salt intake for adults?	80 (15.3)	108 (20.7)	334 (64.0)
What are the criteria for diagnosing hypertension?	178 (34.1)	143 (27.4)	201 (38.5)
Is high salt intake a risk factor for hypertension?	444 (85.1)	30 (5.7)	48 (9.2)
Is high salt intake a risk factor for obesity?	269 (51.5)	86 (16.5)	167 (32.0)
Is excessive salt consumption linked to cardiovascular disease?	356 (68.2)	34 (6.5)	132 (25.3)
Can excessive salt consumption cause kidney problems?	415 (79.5)	18 (3.4)	89 (17.0)
A food is considered high in salt if it contains a percentage starting from.?	55 (10.5)	163 (31.2)	304 (58.2)
Are fast foods high in salt?	441 (84.5)	17 (3.3)	64 (12.3)
Can uncontrolled hypertension lead to serious complications (such as stroke and heart failure)?	396 (75.9)	12 (2.3)	114 (21.8)
Can reducing salt intake help lower blood pressure and improve overall health?	452 (86.6)	10 (1.9)	60 (11.5)

### 3.4 Correlations between HBM constructs, salt-related knowledge, and dietary practices

Pearson correlation analyses revealed several statistically significant associations between HBM constructs, self-efficacy, salt-related knowledge, and dietary practices (Table 4). Salt-related knowledge was positively correlated with perceived susceptibility and severity (*r* = 0.422, *p* < 0.001), perceived risk (*r* = 0.197, *p* < 0.001), perceived benefits (*r* = 0.341, *p* < 0.001), cues to action (*r* = 0.251, *p* < 0.001), likelihood of following the recommended interventions (*r* = 0.316, *p* < 0.001), self-efficacy (*r* = 0.275, *p* < 0.001), and dietary practices (*r* = 0.272, *p* < 0.001), indicating weak to moderate positive associations, with the strongest relationship observed for perceived susceptibility and severity. No significant association was found between knowledge and perceived barriers (*r* = 0.036, *p* = 0.418). Age demonstrated weak positive correlations with perceived risk (*r* = 0.201, *p* < 0.001), cues to action (*r* = 0.252, *p* < 0.001), and likelihood of following the recommended interventions (*r* = 0.120, *p* = 0.006), while BMI was weakly positively associated with perceived risk (*r* = 0.180, *p* < 0.001), cues to action (*r* = 0.223, *p* < 0.001), and likelihood of following the recommended interventions (*r* = 0.124, *p* = 0.005). Importantly, dietary practices were positively correlated with perceived susceptibility and severity (*r* = 0.140, *p* = 0.001), perceived risk (*r* = 0.201, *p* < 0.001), perceived benefits (*r* = 0.141, *p* = 0.001), cues to action (*r* = 0.234, *p* < 0.001), likelihood of following the recommended interventions (*r* = 0.291, *p* < 0.001), and self-efficacy (*r* = 0.248, *p* < 0.001), with likelihood of following the recommended interventions showing the strongest (though still small-to-moderate) association. Perceived barriers were not significantly associated with dietary practices (*r* = 0.069, *p* = 0.117). Overall, although most correlations were statistically significant, their magnitudes were small to approaching moderate (|*r*| = 0.14–0.42), suggesting modest but meaningful relationships between HBM constructs and salt-related dietary behavior.

**Table 4:** Correlations between HBM constructs, salt-related knowledge, dietary practices, age, and BMI (Pearson correlation test) (*n* = 522).

Variable	Correlation coefficients and significance levels	Perceived susceptibility and severity	Perceived risk	Perceived benefits	Cues to action	Likelihood of following the recommended interventions	Perceived barriers	Self-efficacy	Dietary practices
Salt-related Knowledge	*r*	0.422	0.197	0.341	0.251	0.316	0.036	0.275	0.272
	*p*	0.000**	0.000**	0.000**	0.000**	0.000**	0.418	0.000**	0.000**
Age	*r*	0.086	0.201^**^	0.026	0.252^**^	0.120^**^	−0.001	0.055	−0.042
	*p*	0.050	0.000	0.548	0.000	0.006	0.976	0.208	0.330
BMI	*r*	0.064	0.180^**^	0.012	0.223^**^	0.124^**^	0.072	0.033	0.070
	*p*	0.142	0.000	0.781	0.000	0.005	0.101	0.462	0.111
Dietary practices	*r*	0.140^**^	0.201^**^	0.141^**^	0.234^**^	0.291^**^	0.069	0.248^**^	-----
	*p*	0.001	0.000	0.001	0.000	0.000	0.117	0.000	-----

*r*, Correlation Coefficient; *p*, *p*-value. *Correlation is significant at the 0.05 level (2-tailed), **Correlation is significant at the 0.01 level (2-tailed).

### 3.5 Differences in HBM constructs, salt-related knowledge, and dietary practices by binary personal characteristics

Table 5 shows differences in HBM constructs, salt-related knowledge, and dietary practices by binary personal characteristics. Overall, mean scores for HBM constructs ranged from moderate to relatively high (approximately 2.7 to 4.0 out of 5). Perceived benefits had the highest scores (e.g., males: 3.98; females: 3.94), indicating a high level of agreement, while perceived risk was comparatively lower (e.g., 2.78–2.84), reflecting moderate perception. Cues to action and likelihood of following recommendations were moderate to high (around 3.0–3.5). Perceived barriers and self-efficacy were also moderate (around 3.1–3.3). Dietary practices showed lower scores (around 2.66–2.82), suggesting suboptimal adherence to salt reduction behaviors despite relatively favorable perceptions.

**Table 5:** Differences in HBM constructs, salt-related knowledge, and dietary practices by binary personal characteristics of the studied population (*N* = 522).

Variable	Category	Perceived susceptibility and severity	Perceived risk	Perceived benefits	Cues to action	Likelihood of following the recommended interventions	Perceived barriers	Self-efficacy	Salt-related knowledge Min (10) Max (20)	Dietary practices
Gender	Male	3.65	2.84	3.98	3.06	3.37	3.15	3.17	15.23	2.66
	Female	3.74	2.78	3.94	3.11	3.41	3.11	3.27	16.16	2.76
	*t*	−1.013	0.78	0.37	−0.66	−0.44	0.53	−1.03	−4.37	−1.79
	*p*	0.311	0.43	0.71	0.51	0.66	0.59	0.306	0.000*	0.073
	CI	−0.25–0.08	−0.09–0.22	−0.15–0.22	−0.22–0.11	−0.21–0.13	−0.11–0.19	−0.28–0.09	−1.34- -0.51	−0.23–0.01
Marital status	Unmarried	3.63	2.69	3.89	2.91	3.30	3.13	3.23	15.82	2.78
	Married	3.77	2.87	3.99	3.23	3.47	3.11	3.25	15.98	2.71
	*t*	1.88	2.45	1.03	4.43	2.10	−0.19	0.20	0.82	−1.29
	*p*	0.06	0.02*	0.31	0.000*	0.036*	0.85	0.84	0.41	0.198
	CI	−0.01–0.29	0.035–0.317	−0.08–0.26	0.18–0.71	0.01–0.32	−0.15–0.12	−0.15–0.18	−0.22–0.54	−0.17–0.04
History of personal hypertension	Yes	3.71	2.69	3.94	2.98	3.36	3.10	3.24	15.95	2.71
	No	3.72	3.184	3.99	3.52	3.53	3.19	3.26	15.90	2.82
	*t*	0.13	5.96	0.44	6.33	1.85	1.01	0.21	0.24	1.62
	*p*	0.901	0.000*	0.66	0.000*	0.066	0.31	0.82	0.81	0.10
	CI	−0.17–0.19	0.332–0.661	−0.16–0.25	0.38–0.71	−0.01–0.36	−0.08–0.25	−0.17–0.22	−0.39–0.51	−0.02–0.23

*p*, probability value; *t*, t-test; CI, confidence interval; *statistically significant result, a *p*-value of <0.05 is considered statistically significant.

Gender differences were minimal across most constructs; however, females had significantly higher salt-related knowledge scores than males (16.16 vs. 15.23 out of 20, *p* < 0.001). Married participants reported significantly higher perceived risk (2.87 vs. 2.69), cues to action (3.23 vs. 2.91), and likelihood of following recommendations (3.47 vs. 3.30), indicating moderate to high engagement (*p* < 0.05). Participants without hypertension showed significantly higher perceived risk (3.18 vs. 2.69) and cues to action (3.52 vs. 2.98), both reflecting moderate-to-high levels (*p* < 0.001).

### 3.6 Differences in HBM constructs, salt-related knowledge, and dietary practices according to sociodemographic characteristics

Table 6 presents the mean scores of HBM constructs, salt-related knowledge, and dietary practices across multiple demographic characteristics. Table 6 shows that HBM construct scores are generally moderate to high (≈2.6–4.1/5), with perceived benefits the highest (around 3.9–4.2/5) and perceived risk moderate (≈2.6–3.2/5). Cues to action, self-efficacy, and likelihood of adherence are mostly around 3.0–3.6/5. Dietary practices remain lower across all groups (≈2.6–2.9/5), indicating suboptimal salt-reduction behavior despite moderate to good knowledge (≈14.5–16.5/20).

**Table 6:** Comparisons of HBM constructs, knowledge, and dietary practices across multiple demographic (mean ± SD, 95% CI) in the studied population (*N* = 522).

Variable	Category	Perceived susceptibility and severity	Perceived risk	Perceived benefits	Cues to action	Likelihood of following the recommended interventions	Perceived barriers	Self-efficacy	Knowledge to salt min(10) max(20)	Dietary practices
Age (years)	18–29 *N* = 192	3.59 ± 0.89 (3.46–3.73)	2.61 ± 0.78 (2.496–2.732)	3.91 ± 1.05 (3.75–4.07)	2.79 ± 0.84 2.67–2.92	3.25 ± 0.97 (3.10–3.39)	3.08 ± 0.85 (2.96–3.21)	3.12 ± 0.99 (2.97–3.27)	15.82 ± 2.34 (15.46–16.17)	2.76 ± 0.61 (2.674–2.858)
	30–39 *N* = 126	3.83 ± 0.75 (3.703–3.966)	2.82 ± 0.79 (2.68–2.96)	4.01 ± 0.90, (3.851–4.170)	3.22 ± 0.74 (3.09–3.36)	3.59 ± 0.72 (3.46–3.72)	3.11 ± 0.78 (2.98–3.25)	3.49 ± 0.80 (3.35–3.63)	16.40 ± 1.98 (16.05–16.75)	2.81 ± 0.59 (2.70–2.91)
	40–49 *N* = 114	3.73 ± 0.82 (3.58–3.88)	2.88 ± 0.81 (2.71–3.007)	3.96 ± 0.89 (3.80–4.13)	3.27 ± 0.76 (3.12–3.41)	3.46 ± 0.83 (3.31–3.61)	3.24 ± 0.74 (3.10–3.37)	3.21 ± 0.947 (3.03–3.38)	15.49 ± 2.26 (15.07–15.91)	2.68 ± 0.62 (2.56–2.79)
	50–59 *N* = 90	3.87 ± 0.63 (3.59–4.14)	3.20 ± 0.91 (2.81–3.59)	4.04 ± 0.61 (3.78–4.31)	3.30 ± 0.85 (2.94–3.67)	3.57 ± 0.97 (3.15–3.98)	2.96 ± 0.51 (2.74–3.18)	3.43 ± 0.97 (3.01–3.86)	16.26 ± 2.07 (15.36–17.16)	2.76 ± 0.129 (2.49–3.02)
	F	1.417	4.217	0.309	8.325	3.259	0.779	2.831	2.447	1.017
	*p*	0.216	0.001***	0.907	0.000***	0.007***	0.565	0.016**	0.033*	0.407
History of family hypertension	Yes *N* = 384	3.84 ± 0.73 (3.77–3.92)	2.93 ± 0.78 (2.85–3.01)	4.07 ± 0.85 (3.98–4.16)	3.21 ± 0.81 (3.13–3.28)	3.48 ± 0.83 (3.39–3.56)	3.19 ± 0.75 (3.12–3.27)	3.32 ± 0.92 (3.23–3.41)	16.24 ± 1.98 (16.04–16.44)	2.80 ± 0.58 (2.74–2.86)
	No *N* = 86	3.44 ± 1.01 (3.23–3.66)	2.35 ± 0.81 (2.17–2.52)	3.68 ± 1.15 (3.43–3.93)	2.73 ± 0.86 (2.54–2.91)	3.22 ± 0.98 (3.01–3.43)	2.94 ± 0.85 (2.76–3.12)	3.08 ± 1.05 (2.86–3.31)	15.29 ± 2.23, (14.81–15.77)	2.58 ± 0.61, (2.45–2.71)
	I Do not Remember *N* = 52	3.21 ± 0.98 (2.94–3.49)	2.52 ± 0.74 (2.32–2.73)	3.48 ± 1.184 (3.16–3.82)	2.88 ± 0.77 (2.67–3.09)	3.07 ± 0.94, (2.81–3.34)	2.87 ± 0.85 (2.63–3.11)	2.98 ± 0.89 (2.73–3.23)	14.50 ± 2.60 (13.78–15.22)	2.533 ± 0.67 (2.35–2.72)
	F	19.64	23.11	13.03	14.18	7.15	6.79	4.50	20.476	8.11
	*p*	0.000***	0.000***	0.000***	0.000***	0.001***	0.001***	0.012**	0.000***	0.000***
Received advice from doctors	Yes (*n* = 163)	3.82 ± 0.71 (3.71–3.93)	3.09 ± 0.73 (2.98–3.20)	4.07 ± 0.87 (3.93–4.20)	3.52 ± 0.71 (3.41–3.63)	3.55 ± 0.86 (3.41–3.68)	3.23 ± 0.75 (3.11–3.35)	3.25 ± 0.94 (3.11–3.39)	16.10 ± 2.03 (15.79–16.42)	2.83 ± 0.67 (2.73–2.93)
	No (302)	3.71 ± 0.88 (3.61–3.81)	2.64 ± 0.81 (2.55–2.73)	3.94 ± 0.99 (3.83–4.05)	2.87 ± 0.83 (2.78–2.96)	3.32 ± 0.91 3.22–3.43	3.08 ± 0.79 (2.99–3.17)	3.25 ± 0.97 (3.14–3.36)	15.90 ± 2.15 (15.66–16.14)	2.69 ± 0.57 (2.625–2.755)
	I Do not Remember (57)	3.451 ± 0.88 (3.22–3.69)	2.76 ± 0.81 (2.55–2.98)	3.66 ± 0.99 (3.39–3.93)	3.09 ± 0.72 (2.89–3.27)	3.37 ± 0.74 (3.17–3.57)	3.00 ± 0.83 (2.78–3.22)	3.21 ± 0.81 (3.00–3.42)	(15.42 ± 2.55, 14.74–16.10)	2.72 ± 0.60 (2.563–2.882)
	F	4.086	18.068	3.851	35.754	3.522	2.611	0.045	2.120	2.792
	*p*	0.017**	0.000***	0.022**	0.000***	0.030**	0.074	0.956	0.121	0.062
Received advice from family/friends	Yes 306	3.76 ± 0.790 (3.67–3.85)	2.914 ± 0.80 (2.82–3.01)	4.01 ± 0.91 (3.91–4.11)	3.29 ± 0.77 (3.21–3.38)	3.45 ± 0.82 (3.36–3.55)	3.19 ± 0.76 (3.11–3.28)	3.25 ± 0.92 (3.15–3.35)	16.01 ± 2.08 (15.78–16.25)	2.79 ± 0.62 (2.72–2.86)
	No 183	3.69 ± 0.90 (3.55–3.82)	2.63 ± 0.81 (2.51–2.74)	3.89 ± 1.03 (3.74–4.04)	2.81 ± 0.84 (2.69–2.94)	3.34 ± 0.97 (3.19–3.48)	3.04 ± 0.83 2.91–3.16	3.26 ± 1.00 (3.11–3.41)	15.94 ± 2.12 (15.63–16.25)	2.67 ± 0.58 2.58–2.75
	I Do not Remember *N* = 33	3.46 ± 0.89 (3.15–3.78)	2.61 ± 0.73 (2.36–2.86)	3.72 ± 1.00 (3.36–4.07)	2.82 ± 0.79 (2.54–3.10)	3.19 ± 0.81 (2.89–3.48)	2.93 ± 0.73 (2.67–3.19)	3.10 ± 0.85 (2.80–3.40)	14.82 ± 2.84 (13.81–15.83)	2.63 ± 0.64 (2.40–2.86)
	F	1.972	8.389	1.914	22.504	1.995	3.328	0.413	4.620	2.870
	*p*	0.140	0.000***	0.149	0.000***	0.137	0.037**	0.662	0.010**	0.058
Seen media advertising about salt reduction	Yes *N* = 344	3.79 ± 0.81 (3.71–3.89)	2.83 ± 0.80 (2.75–2.92)	4.00 ± 0.95 (3.90–4.10)	3.21 ± 0.82 (3.13–3.29)	3.46 ± 0.89 (3.37–3.56)	3.11 ± 0.76 (3.03–3.19)	3.31 ± 0.98 (3.21–3.41)	16.29 ± 1.94 (16.09–16.50)	2.79 ± 0.61 (2.73–2.85)
	No *N* = 112	3.49 ± 0.98 (3.31–3.67)	2.63 ± 0.87 (2.47–2.79)	3.85 ± 1.08 (3.65–4.05)	2.84 ± 0.88 (2.68–3.01)	3.28 ± 0.92 (3.11–3.45)	3.14 ± 0.90 (2.97–3.31)	3.05 ± 0.91 (2.88–3.22)	15.05 ± 2.51 (14.58–15.52)	2.69 ± 0.656 (2.56–2.81)
	I Do not Remember *N* = 66	3.64 ± 0.67 (3.47–3.81)	2.87 ± 0.74 (2.69–3.05)	3.84 ± 0.75 (3.66–4.03)	2.93 ± 0.72 (2.75–3.10)	3.26 ± 0.74 (3.08–3.45)	3.17 ± 0.73 (2.99–3.35)	3.24 ± 0.76 (3.06–3.43)	15.39 ± 2.18 (14.86–15.93)	2.55 ± 0.48 (2.43–2.66)
	F	6.169	2.902	1.501	10.233	2.709	0.215	3.371	16.918	4.949
	*p*	0.002***	0.056	0.224	0.000***	0.068	0.807	0.035**	0.000***	0.007***
Occupation	Private Sector *N* = 101	3.83 ± 0.63 (3.70–3.95)	2.87 ± 0.79 (2.71–3.03)	4.21 ± 0.77 (4.06–4.36)	3.37 ± 0.67 (3.24–3.50)	3.61 ± 0.78 (3.45–3.76)	3.24 ± 0.78 (3.08–3.39)	3.37 ± 0.84 (3.21–3.54)	15.93 ± 2.13 (15.51–16.35)	2.86 ± 0.60 (2.74–2.97)
	Public Sector *N* = 132	3.74 ± 0.96 (3.57–3.90)	2.94 ± 0.77 (2.81–3.07)	3.95 ± 1.02 (3.78–4.13)	3.09 ± 0.85 (2.95–3.24)	3.42 ± 0.91 (3.26–3.56)	3.11 ± 0.84 (2.96–3.25)	3.24 ± 0.96 (3.08–3.41)	15.95 ± 2.13 (15.58–16.31)	2.71 ± 0.66 (2.59–2.83)
	Self-Employed *N* = 16	3.68 ± 0.85 (3.23–4.14)	2.54 ± 0.81 (2.11–2.97)	3.75 ± 1.02 (3.21–4.29)	3.24 ± 0.73 (2.85–3.63)	3.31 ± 0.86 (2.86–3.77)	3.07 ± 0.62 (2.74–3.39)	3.52 ± 1.12 (2.93–4.12)	15.81 ± 2.43 (14.52–17.11)	2.72 ± 0.58 (2.42–3.03)
	Student *N* = 116	3.61 ± 0.91 (3.44–3.77)	2.63 ± 0.85 (2.47–2.78)	3.87 ± 1.08 (3.67–4.07)	2.77 ± 0.88 (2.61–2.94)	3.21 ± 0.99 (3.03–3.39)	3.06 ± 0.89 (2.89–3.23)	3.08 ± 1.07 (2.89–3.28)	15.92 ± 2.37 (15.49–16.36)	2.76 ± 0.63 (2.64–2.87)
	None *N* = 157	3.69 ± 0.79 (3.57–3.82)	2.76 ± 0.80 (2.64–2.89)	3.85 ± 0.89 (3.71–3.99)	3.14 ± 0.82 (3.01–3.27)	3.39 ± 0.80 (3.27–3.52)	3.10 ± 0.66 (2.99–3.21)	3.25 ± 0.86 (3.11–3.38)	15.87 ± 2.06, (15.55–16.20)	2.67 ± 0.54 (2.58–2.75)
	F	0.979	3.045	2.602	7.686	2.836	0.764	1.605	0.032	1.547
	*p*	0.418	0.017*	0.035*	0.000***	0.024*	0.549	0.172	0.998	0.187
Region	North *N* = 92	3.39 ± 0.97 (3.19–3.59)	2.55 ± 0.83 (2.37–2.72)	3.59 ± 1.15 (3.36–3.83)	2.98 ± 0.93 (2.78–3.17)	3.29 ± 0.94 (3.09–3.49)	3.05 ± 0.83 (2.88–3.22)	3.21 ± 0.98 (3.01–3.41)	15.61 ± 2.14 (15.16–16.05)	2.68 ± 0.62 (2.56–2.81)
	South *N* = 50	3.60 ± 0.89 (3.34–3.85)	2.61 ± 0.73 (2.40–2.82)	3.71 ± 1.07 (3.401–4.013)	2.95 ± 0.765 (2.74–3.17)	3.20 ± 0.91 (2.94–3.46)	3.06 ± 0.87 (2.81–3.31)	3.07 ± 0.99 (2.79–3.36)	15.56 ± 2.30 (14.91–16.21)	2.63 ± 0.69 (2.43–2.83)
	Central *N* = 63	3.79 ± 0.69 (3.62–3.97)	2.84 ± 0.72 (2.66–3.02)	3.92 ± 0.79 (3.72–4.12)	3.00 ± 0.72 (2.82–3.18)	3.40 ± 0.69 (3.23–3.58)	3.05 ± 0.69 (2.87–3.22)	3.25 ± 0.77 (3.06–3.45)	15.98 ± 2.37 (15.39–16.58)	2.76 ± 0.57 (2.62–2.91)
	East *N* = 58	3.78 ± 0.71 (3.59–3.97)	2.85 ± 0.85 (2.63–3.07)	4.09 ± 0.72 (3.89–4.28)	3.43 ± 0.77 (3.22–3.63)	3.65 ± 0.72 (3.46–3.84)	3.30 ± 0.70 (3.12–3.49)	3.44 ± 0.71 (3.25–3.62)	16.36 ± 1.72 (15.91–16.82)	2.93 ± 0.65 (2.76–3.10)
	West *N* = 259	3.81 ± 0.81 (3.71–3.91)	2.89 ± 0.81 (2.79–2.99)	4.10 ± 0.91 (3.98–4.21)	3.12 ± 0.83 (3.01–3.22)	3.418 ± 0.91 (3.31–3.53)	3.13 ± 0.79 (3.04–3.23)	3.25 ± 1.00 (3.12–3.37)	15.97 ± 2.18 (15.70–16.24)	2.73 ± 0.58 (2.66–2.79)
	F	4.892	3.951	6.073	3.385	2.178	1.210	1.045	1.475	2.086
	*p*	0.001***	0.004**	0.000***	0.010*	0.070	0.306	0.383	0.208	0.081
Income	Less than 5,000 *N* = 232	3.65 ± 0.86 (3.54–3.76)	2.72 ± 0.84 2.61–2.83	3.85 ± 0.98 (3.2–3.97)	3.02 ± 0.87 (2.91–3.13)	3.33 ± 0.884 (3.212–3.441)	(3.102 ± 0.797)2.999–3.205	(3.223 ± 0.951)3.100–3.346	15.78 ± 2.26 (15.48–16.07)	2.69 ± 0.62 2.62–2.77
	From 5,000 to 10,000 *N* = 129	3.65 ± 0.88 (3.49–3.80)	2.71 ± 0.77 (2.57–2.84)	3.94 ± 1.08 (3.75–4.13)	3.07 ± 0.84 (2.92–3.2)	3.39 ± 0.95 (3.23–3.56)	3.10 ± 0.82 (2.95–3.24)	3.28 ± 0.97 (3.11–3.45)	16.31 ± 2.06 (15.95–16.67)	2.75 ± 0.58 (2.65–2.85)
	From 11,000 to 20,000 *N* = 107	3.84 ± 0.74 (3.69–3.98)	3.00 ± 0.79 (2.85–3.16)	4.11 ± 0.77 (3.96–4.26)	3.29 ± 0.77 (3.15–3.44)	3.578 ± 0.76 (3.43–3.72)	3.17 ± 0.73 (0.03–3.31)	3.40 ± 0.88 (3.24–3.57)	15.67 ± 2.28, 15.24–16.11	2.79 ± 0.62 (2.68–2.92)
	More than 20,000 *N* = 54	3.89 ± 0.81, (3.67–4.11)	2.91 ± 0.78 (2.69–3.12)	4.09 ± 0.91 (3.85–4.35)	3.09 ± 0.76 (2.88–3.29)	3.36 ± 0.87 (3.12–3.59)	3.14 ± 0.75 (2.94–3.35)	2.93 ± 0.93 (2.68–3.18)	16.02 ± 1.63 (15.57–16.46)	2.77 ± 0.62 (2.60–2.94)
	F	2.354	3.956	2.394	2.810	2.068	0.230	3.149	2.253	0.749
	*p*	0.071	0.008**	0.068	0.039*	0.104	0.875	0.025*	0.081	0.523
Education level	Primary Education *N* = 9	3.17 ± 0.93 (2.45–3.88)	2.47 ± 0.59 (2.01–2.92)	3.04 ± 0.82 (2.40–3.67)	3.20 ± 0.65 (2.70–3.71)	3.02 ± 0.65 (2.51–3.52)	2.96 ± 0.57 (2.52–3.40)	2.85 ± 0.60 (2.39–3.32)	13.78 ± 2.99 (11.48–16.08)	2.30 ± 0.59 (1.84–2.76)
	Secondary Education *N* = 97	3.57 ± 0.88 (3.39–3.75)	2.81 ± 0.86 (2.63–2.98)	3.85 ± 1.08 (3.63–4.07)	3.18 ± 0.91 (2.99–3.36)	3.30 ± 0.94 (3.12–3.49)	3.08 ± 0.80 (2.92–3.24)	3.18 ± 0.94 (2.99–3.37)	15.88 ± 2.04 (15.46–16.29)	2.70 ± 0.67 (2.56–2.84)
	Undergraduate *N* = 327	3.68 ± 0.84 (3.59–3.77)	2.73 ± 0.81 (2.64–2.82)	3.94 ± 0.96 (3.84–4.05)	3.06 ± 0.83 (2.97–3.15)	3.39 ± 0.89 (3.30–3.49)	3.13 ± 0.80 (3.05–3.22)	3.23 ± 0.96 (3.12–3.33)	15.83 ± 2.17 (15.60–16.07)	(2.73 ± 0.59, 2.66–2.79)
	Postgraduate Studies *N* = 89	4.04 ± 0.70, (3.90–4.19)	3.05 ± 0.72 (2.90–3.20)	4.18 ± 0.75 (4.02–4.33)	(3.13 ± 0.78, 2.96–3.29)	(3.55 ± 0.75, 3.39–3.71)	3.13 ± 0.72 (2.98–3.29)	3.42 ± 0.89 (3.24–3.61)	16.45 ± 2.06 (16.02–16.88)	(2.86 ± 0.59, 2.73–2.98)
	F	7.245	4.212	4.353	0.568	1.834	0.247	1.801	4.999	2.937
	*p*	0.000***	0.006***	0.003***	0.637	0.140	0.863	0.146	0.002***	0.033**
BMI category	Underweight *N* = 32	3.71 ± 1.00 (3.35–4.07)	2.55 ± 0.73 (2.29–2.81)	4.08 ± 1.05 (3.70–4.46)	2.76 ± 0.86 (2.45–3.07)	3.15 ± 0.88, (2.84–3.47)	3.07 ± 0.87 (2.78–3.36)	3.34 ± 1.09 (2.95–3.73)	16.22 ± 2.25, (15.41–17.03)	2.70 ± 0.56 (2.49–2.90)
	Normal Weight *N* = 184	1.65 ± 0.81 (3.53–3.77)	2.64 ± 0.74 (2.53–2.75)	3.91 ± 0.90, (3.78–4.05)	2.89 ± 0.77 (2.78–3.01)	3.27 ± 0.86 (3.15–3.40)	3.07 ± 0.79 (2.95–3.18)	3.16 ± 0.94 (3.02–3.29)	15.85 ± 2.25 (15.52–16.18)	2.68 ± 0.57 (2.59–2.76)
	Overweight *N* = 166	3.70 ± 0.84 (3.57–3.83)	2.86 ± 0.79 (2.74–2.98)	3.91 ± 0.95 (3.76–4.06)	3.19 ± 0.79 (3.08–3.32)	3.49 ± 0.81 (3.37–3.62)	3.10 ± 0.77 (2.98–3.22)	3.29 ± 0.85 (3.16–3.42)	15.77 ± 2.15 (15.43–16.10)	2.76 ± 0.61 (2.67–2.86)
	Obese *N* = 140	3.81 ± 0.83 (3.67–3.94)	2.97 ± 0.89 (2.82–3.12)	4.01 ± 1.02 (3.83–4.18)	3.31 ± 0.88 (3.16–3.46)	3.49 ± 0.96 (3.33–3.657)	3.22 ± 0.77 (3.09–3.35)	3.28 ± 1.02 (3.11–3.45)	16.10 ± 2.05 (15.76–16.44)	2.79 ± 0.65 (2.68–2.89)
	F	0.999	5.946	0.530	9.597	3.257	1.112	0.809	0.873	1.024
	*p*	0.393	0.001***	0.662	0.000***	0.021**	0.344	0.489	0.455	0.382

Significant differences were observed by age, with older participants (50–59 years) showing higher perceived risk (3.20), cues to action (3.30), and likelihood (3.57; *p* < 0.05). A family history of hypertension was strongly associated with higher scores across all constructs, knowledge, and practices (*p* < 0.001). Receiving advice from doctors, family/friends, and exposure to media were all associated with higher perceived risk, cues to action, and knowledge (e.g., knowledge 16.29 vs. 15.05 with media exposure). Education also showed a clear gradient, with postgraduate participants reporting higher perceived benefits (4.18/5) and knowledge (16.45/20). Overall, although perceptions and knowledge are favorable, dietary practices remain comparatively low, highlighting a gap between awareness and behavior.

## 4 Discussion

This study examined salt-related knowledge, habitual dietary practices, and psychosocial determinants of salt-reduction behaviors among 522 Saudi adults using an extended HBM. Findings reveal a complex interaction between cognitive factors, sociodemographic characteristics, and actual dietary behaviors, with implications for public health interventions targeting the reduction of salt intake. While general knowledge of salt-related health risks was moderate to high, technical knowledge on recommended intake, labeling thresholds, and hypertension criteria remained limited. Habitual dietary practices included unrestricted salt use during cooking, frequent consumption of processed foods, and low adoption of active salt-reduction behaviors.

Participants exhibited high knowledge of the health risks of excessive salt intake, with 85.1% recognizing hypertension risk and 79.5% kidney-related risks, consistent with prior Saudi studies (14, 38). However, knowledge of actionable recommendations was low: only 15.3% correctly identified the World Health Organization daily limit, and label-reading knowledge was limited. This pattern mirrors findings in Abha and Madinah, suggesting that current public health messaging effectively raises awareness but fails to convey practical guidance for behavior change (14, 26, 38). Women and participants with higher education showed greater knowledge, highlighting the role of gender and educational attainment in health literacy (38, 39). The salt use during cooking was high (55.2% always, 25.3% often), whereas table salt addition was less frequent. Frequent consumption of processed foods and fast foods (66–75%) poses additional risks, reflecting a shift toward Western dietary patterns (40). Active salt-reduction behaviors, including label reading (37.6% engaged) and using substitutes (23.2%), were limited. These findings underscore the persistent knowledge–behavior gap, which aligns with previous Saudi studies reporting low translation of knowledge into practice (14, 26, 38).

Most HBM constructs were positively associated with dietary practices, particularly likelihood of following recommended interventions (*r* = 0.291), self-efficacy (*r* = 0.248), and cues to action (*r* = 0.234), supporting the utility of the extended HBM in predicting behavior (19, 24, 41). Perceived barriers were not significantly correlated with behavior, possibly due to high educational attainment and moderate barrier perception (23). External cues, including advice from healthcare providers, family, and media exposure, significantly enhanced perceived risk, benefits, and behavioral intention, consistent with the Theory of Planned Behavior and prior evidence (42, 43). Older adults, participants with a family history of hypertension, women, and those with higher education exhibited higher knowledge, HBM scores, and healthier behaviors, reflecting accumulated health experience and motivation Regional variations suggest that exposure to health messages and local dietary practices influence perceptions and behaviors (44).

Despite moderate to high cognitive readiness, dietary practices remained suboptimal (≈2.6–2.9/5), highlighting the intention–behavior gap (45). Barriers include taste preferences, cultural meal patterns, limited availability of low-sodium options, and insufficient practical skills in label reading and restaurant requests. Multifaceted interventions addressing both individual behaviors and food environments are needed to achieve meaningful reductions in salt intake.

### 4.1 Strengths and limitations

Strengths include the large, regionally diverse sample, use of a translated and validated culturally adapted instrument for the study, and application of an extended HBM framework. Limitations include the cross-sectional design (46), reliance on self-reported dietary practices (47), convenience sampling (48), and absence of objective sodium measures (47). Several additional sample characteristics warrant consideration when interpreting the findings. First, the sample was characterized by a strong female predominance, which may limit the representativeness of findings for male adults and introduce potential gender-related participation bias in health behavior research (49). Second, the disproportionately high educational attainment of participants and the use of online recruitment through social media platforms may have introduced digital access and self-selection biases, potentially underrepresenting individuals with lower educational levels or limited internet access, thereby constraining the generalizability of the findings (48, 50).

### 4.2 Implications for public health

It is recommended that public health strategies in Saudi Arabia could consider focusing on: (1) improving actionable knowledge through clear, practical messaging; (2) building skills and self-efficacy in label reading and low-salt cooking; (3) leveraging healthcare providers, family, and media as cues to action; (4) targeting interventions to specific demographic subgroups; and (5) implementing structural and policy measures, including sodium reformulation, front-of-pack labeling, and restrictions on high-salt food marketing (5, 17, 51). In this context, the promotion of potassium-containing lower-sodium salt substitutes represents an additional evidence-based strategy, consistent with the 2025 WHO guideline recommending their use as a cost-effective approach to reducing sodium intake and lowering cardiovascular disease risk. However, such substitutes are not appropriate for everyone; they are contraindicated in individuals with kidney impairment or other conditions that may compromise potassium excretion, and their promotion should therefore be accompanied by appropriate clinical guidance (52).

## 5 Conclusion

Saudi adults demonstrate moderate to high awareness of salt-related health risks but limited technical knowledge and low adoption of salt-reduction behaviors. The findings suggest that behavioral intention, self-efficacy, and cues to action are associated with more favorable salt-reduction practices. Targeted, multifaceted interventions addressing cognitive, skill-based, and environmental determinants are essential to reduce salt intake, prevent hypertension, and support national and global health objectives.

## Data Availability

The raw data supporting the conclusions of this article will be made available by the authors, without undue reservation.
